# Supplementary material for: Effect of Acute Enriched Environment Exposure on Brain Oscillations and Activation of the Translation Initiation Factor 4E-BPs at Synapses across Wakefulness and Sleep in Rats
Source: Cells. 2023 Sep 20;12(18):2320. doi: 10.3390/cells12182320 (PMC10528220; doi:10.3390/cells12182320)
Supplement: Supplementary file 1 [file cells-12-02320-s001.zip › cells-2562234-supplementary.pdf]

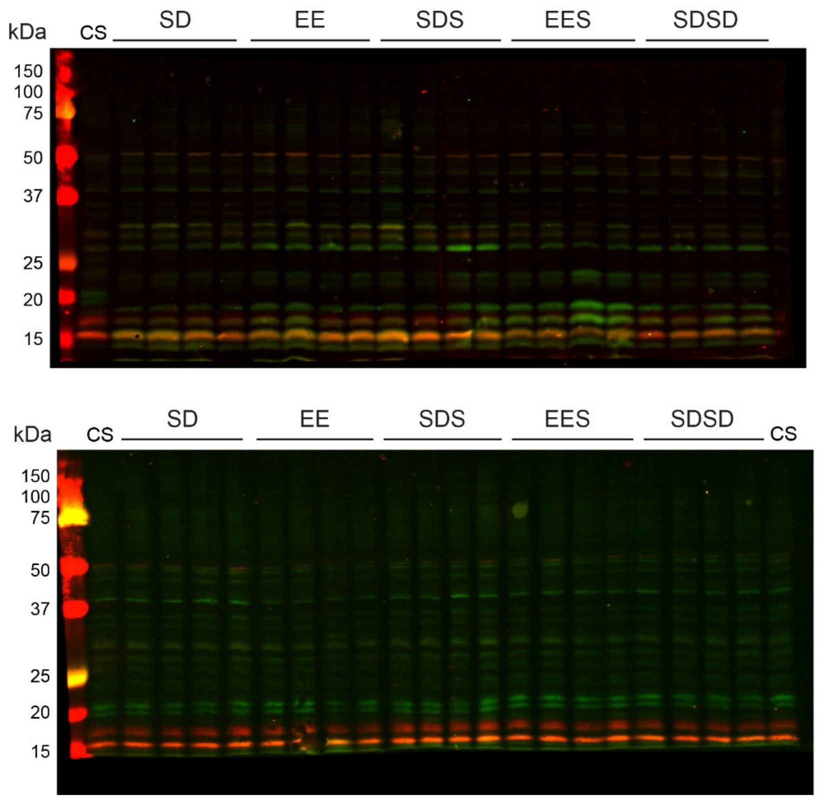

Figure S1. Original Western Blot images. Uncropped and unadjusted images of membranes used in **Figure 4** (upper blot) and **Figure S3** (lower blot). Groups are the same as in main figures. CS: Control sample.

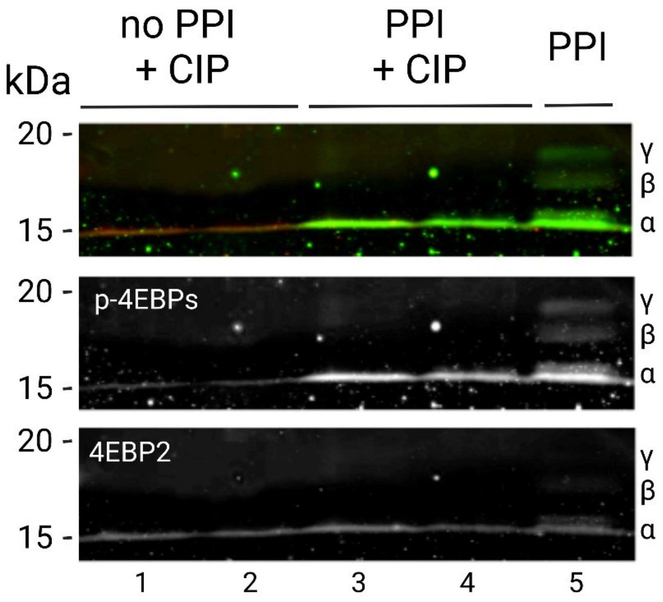

Figure S2. Validation of 4E-BPs phosphorylated forms. Western Blot of cortical extracts probed with antibodies detecting Phospo-4E-BPs (Thr37/46) shown in green and 4E-BP2 protein in red. The individual channels are shown separately below. **Lane 5**: phosphorylation profile of a representative sample used in the study (i.e., homogenised in buffer containing Phosphatase inhibitors (PPI)). **Lanes 1-4**: samples treated with Calf-intestinal alkaline phosphatase (CIP) with (Lanes 3 and 4) or without (Lanes 1 and 2) previous PPI treatment (see **Material and Methods**). Note the absence of the phosphorylated (β) and hyperphosporylated (γ) forms under CIP treatment after treatment with PPI and the disappearance of the hypophosporylated form when samples were not pre-treated with PPI. The unphosphorylated protein (4E-BP2 labelling) remain unaffected by phosphatases.

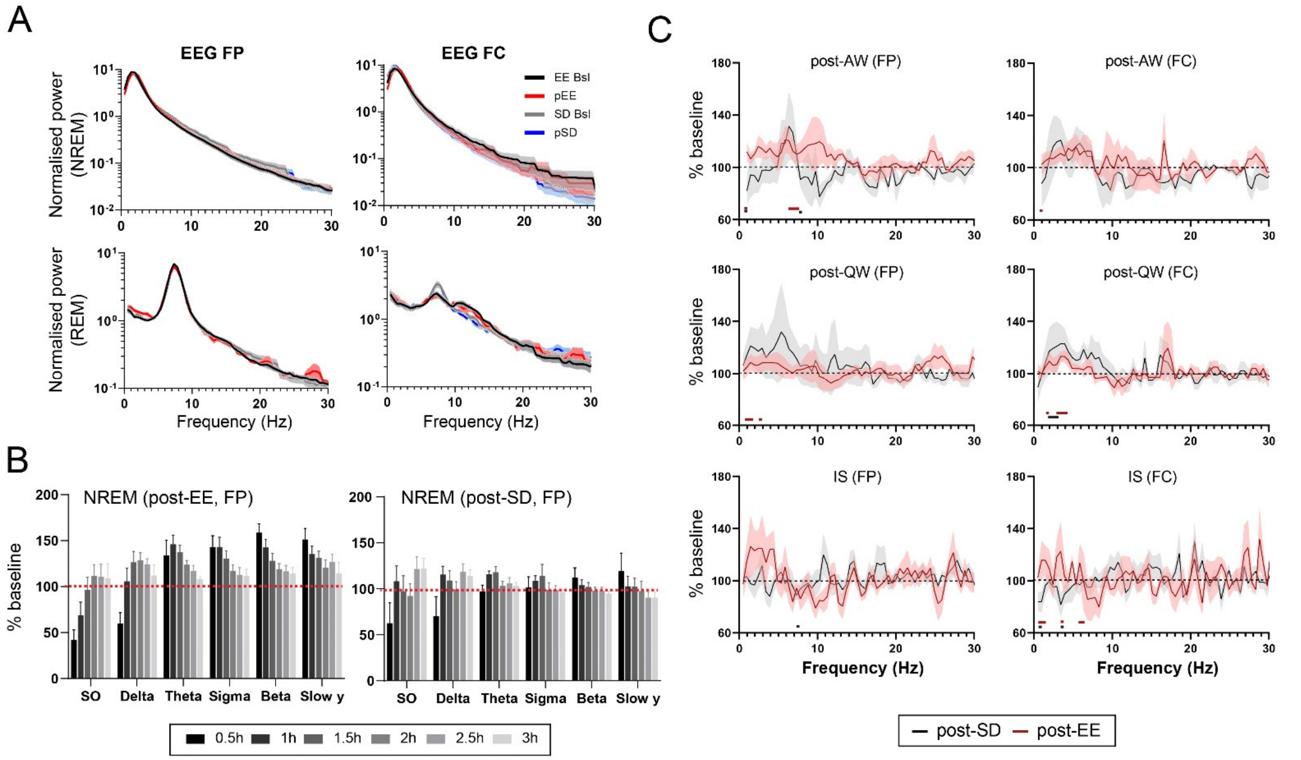

Figure S3. EEG changes during the post-SD/EE rest period. (A) Mean (± SEM) EEG power spectra (normalized to the mean across all frequencies (see Material and Methods) for NREM and REM sleep and both EEGs (FP and FC) separately for both groups (Bsl and post-SD/EE periods) (B) Time course of changes in EEG power (parietal cortex) in all frequency bands (mean ± SEM) in 30-min bins for NREM sleep during the 3-hour rest period after staying awake in an EE or HC. **(C)** Change in mean (± SEM) power density for AW, QW and IS over the 3-hour awake period in EE or HC expressed as % of corresponding baseline values (see **Material and Methods**). The parietal and frontal EEG are shown separately. For clarity, only the presence of significant differences from baseline value are shown underneath the traces. Detailed statistics (Two-Way RM ANOVAs and level of significance for each comparison) can be found in the **Data S1**.

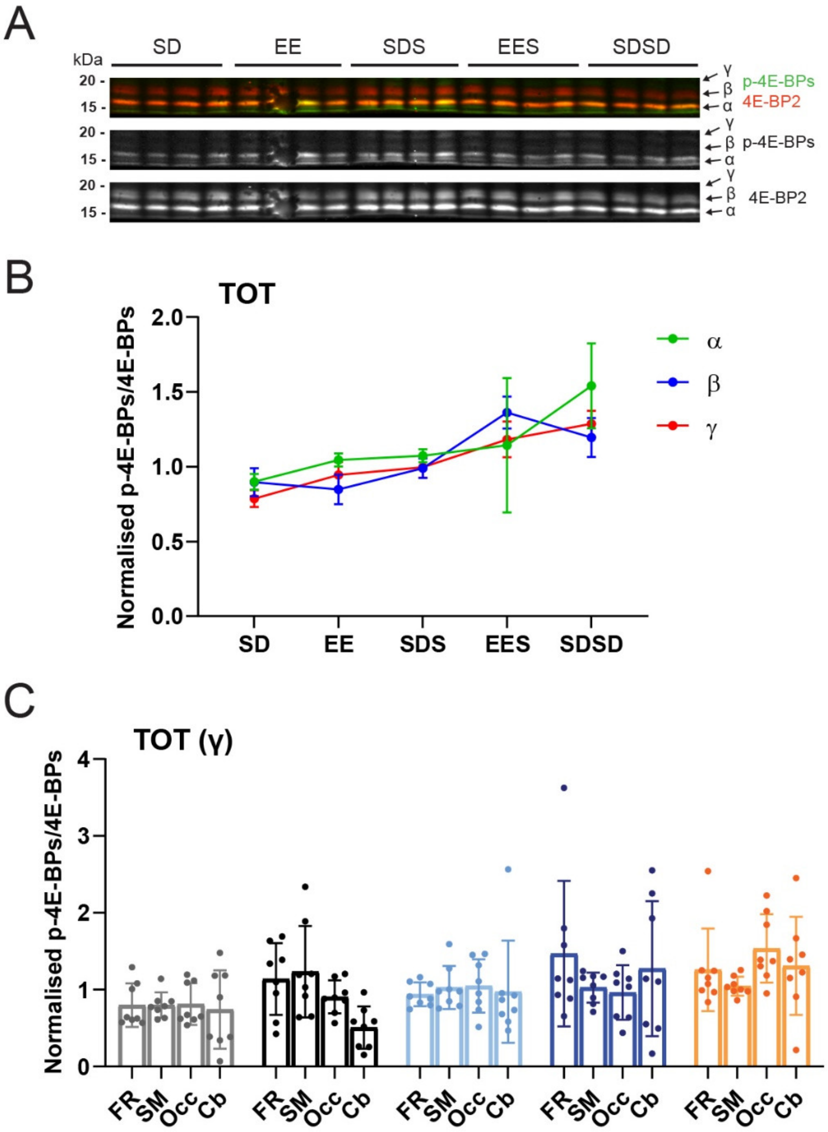

Figure S4. Sleep and experience do not change 4E-BPs phosphorylation in TOT fractions. (A) Representative Western Blot of TOT extracts probed with antibodies detecting Phospo-4E-BPs (Thr37/46) shown in green, 4E-BP2 protein in red and the superimposition of strong signals from either or both channels appears in yellow. The individual channels are shown separately below. The EE group has one samples that did not transfer correctly **(B)** Normalized mean (± SEM) signals from antibodies detecting P-4E-BPs/4EPB2 across groups. **(C)** Distribution of changes of the γ 4E-BPs form in TOT fractions in all 4 brain regions (N = 8/region). A two-way RM ANOVA revealed no effect of groups or brain regions.

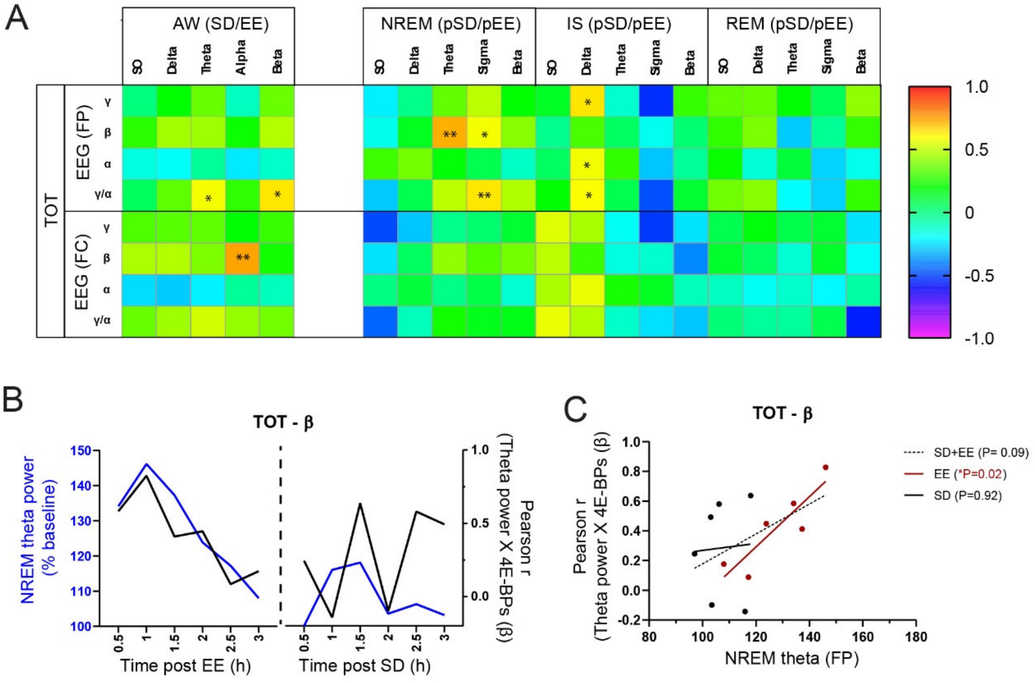

Figure S5. Relation between wake and sleep EEG changes and 4E-BPs measures in whole cellular fraction (TOT). (A) Correlations matrix between changes in frequency band power in AW during the 3 hours of wake (in HC and EE) or sleep (NREM, IS, and REM) during the rest period and changes in 4E-BPs forms (α, β, γ) and conversion index (γ/α ratio). Results are shown for the TOT fraction and separately for each EEG. Correlation coefficients were computed with datapoints from EE and SD groups combined. *P<0.05, **P<0.01, Pearson's. **(B)** Co-variation across the 3 hours post-SD or post-EE (in 30 minutes bins) of correlation strength between NREM theta (blue lines) and 4E-BPs β levels from the EE (*left*) and SD (*right*) group. **(C)** Scatter plot showing the significance of the co-variations shown in (B) for the EE group and SD group.
